# Preoperative estimation of intracranial compliance in symptomatic children with Chiari malformation type 1: impact on outcome and risk of complications

**DOI:** 10.1007/s00701-024-05897-3

**Published:** 2024-01-18

**Authors:** Radek Frič, Eline Bryne, Bogna Warsza, Bernt Johan Due-Tønnessen, Per Kristian Eide

**Affiliations:** 1 Department of Neurosurgery, Oslo University Hospital–Rikshospitalet, P.O. Box 4950, N-0424 Oslo, Norway; 2 Faculty of Medicine, Institute of Clinical Medicine, University of Oslo, Oslo, Norway; 3 Department of Radiology, Oslo University Hospital–Rikshospitalet, Oslo, Norway

**Keywords:** Chiari malformation, Intracranial compliance, Intracranial pressure, Foramen magnum decompression

## Abstract

**Background:**

The role of reduced intracranial compliance (ICC) in the outcome after foramen magnum decompression (FMD) was demonstrated in adults with Chiari malformation Type 1 (CMI). However, similar observations from children treated for CMI are missing.

**Methods:**

We reviewed pediatric cases of CMI referred to FMD between 2006 and 2022. Children with clinical and/or radiological signs suggesting reduced ICC (Group A) underwent overnight measurements of the pulsatile intracranial pressure (ICP): mean ICP wave amplitude (MWA) served as a surrogate marker of ICC. Children with more typical symptoms of CMI (Group B) underwent FMD without preoperative ICC estimation. This study presents the clinical, radiological, and outcome differences between these groups.

**Results:**

Sixty-four children (mean age 11.1 ± 4.3 years) underwent FMD: In Group A (*n* = 30), the finding of reduced ICC as estimated from preoperative ICP measurement resulted in CSF diversion (ventriculoperitoneal shunt) before FMD in 11 children. Two patients required shunt due to complications after FMD (total shunt rate 43%). In Group B (n = 34) treated with FMD without preoperative ICC estimation, five children (15%) required shunting due to complications. In Group A, we found a significantly higher frequency of headache, nausea, fatigue, and dizziness. The outcome assessed by the modified Chicago Chiari Outcome Scale (mean follow-up 83 ± 57 months) was comparable between the groups, but the complication rate after FMD was significantly lower in Group A (7% vs. 32%; *p* = 0.011). The number of procedures (ICP measurement, FMD, shunt, re-do FMD, shunt revisions) was significantly higher in Group A (2.6 ± 0.9 vs. 1.5 ± 1.1 per patient; *p* < 0.001).

**Conclusion:**

In symptomatic children with CMI, the preoperative estimation of ICC from the overnight measurement of pulsatile ICP was more reliable for identifying those with reduced ICC than clinical and radiological assessment alone. When children with abnormally reduced ICC were identified and treated with CSF diversion before FMD, the complication rate was significantly reduced.

## Introduction

More than 130 years after the first systematic pathoanatomical description of abnormal hindbrain herniation without associated dysraphism [39], later denoted as Chiari malformation type 1 (CMI), there is still a lack of understanding as to the etiology and pathophysiology of the condition.

The dynamics of pathological changes typical for CMI is more obvious in children than in adults [46]. In pediatric patients with CMI requiring surgery, the choice of optimal treatment strategy is therefore crucial for a successful outcome and should be based on the correct identification of the underlying cause of tonsillar ectopy [23, 37, 48].

Although foramen magnum decompression (FMD) has been a preferred surgical option for patients with CMI since its original description almost a century ago [40], it may not always address the primary underlying pathophysiological event behind CMI. This applies particularly to cases of CMI associated with craniocervical instability or occult spinal cord tethering, i.e., the pathological conditions that can be relatively easily recognized and addressed. Also CMI secondary to obvious cranial constriction (i.e., craniosynostosis) can be solved by surgical treatment of the primary condition. However, cases associated with or secondary to intracranial hypertension may be difficult to diagnose from clinical and radiological investigations alone.

Intracranial hypertension is associated with reduced intracranial compliance (ICC), i.e., impaired intracranial pressure–volume reserve capacity. We previously identified reduced ICC as an important factor present in a significant proportion of adult patients with CMI [20, 22]. Moreover, it appears that reduced ICC does not automatically normalize after FMD [21, 42], a fact that may result in postoperative complications such as cerebrospinal fluid (CSF) leakage as well as failure to improve after FMD [3, 13]. Identifying the patients with reduced ICC preoperatively is therefore of clinical importance, as many of these patients may benefit from CSF diversion, i.e., the ventriculoperitoneal (VP) shunt, before FMD [12, 24].

However, it is complicated to retrieve information about ICC in a clinically feasible fashion, as it is difficult to disclose reduced ICC from clinical and radiological assessment alone. A direct measurement of ICC requires addition or subtraction of an intracranial volume and is hardly useful in the clinical setting [19, 26]. As a less invasive estimate of ICC retrieved directly from the continuous intracranial pressure (ICP) recording, the mean ICP wave amplitude (MWA) score was found to correlate with direct measurements of ICC, and overnight average scores of MWA < 4–5 mmHg or > 4-–5 mmHg were accompanied with direct measures of normal or reduced ICC, respectively [16, 18, 19]. At our institution, we have therefore utilized metric MWA as a surrogate marker of ICC in different conditions related to disturbed CSF circulation [19].

In symptomatic adult patients with CMI, we have since 2006 used preoperative estimation of ICC from MWA scores as a routine part of preoperative workup [24]. In pediatric patients, however, where the use of invasive ICP monitoring is more questionable, we have followed a more selective strategy: preoperative ICC estimation from the MWA scores was utilized only in cases with clinical and/or radiological signs indicative of reduced ICC (mainly global/diffuse headache of another character than usual in CMI, fatigue, cognitive difficulties, etc., and/or ventriculomegaly or radiological signs of intracranial hypertension). In these children undergoing overnight ICP monitoring, the MWA measures of reduced ICC gave indication to shunt surgery before FMD, while FMD was the first-line treatment in those with normal ICC as well as in children without the need for preoperative ICC estimation.

In this retrospective study, we describe the clinical, radiological, and outcome differences between these two groups of children with CMI treated with FMD, i.e., those with and without preoperative diagnostic estimation of ICC, respectively.

## Methods

The study was approved by Oslo University Hospital (reference number 21/01306) as a quality control study. The regional ethical committee was informed in writing and had no objections to the study.

In this study, we retrospectively reviewed all consecutive cases of children (0–18 years of age) treated surgically with FMD for CMI at the Department of Neurosurgery, Oslo University Hospital-Rikshospitalet, between May 2006 and November 2022.

**Fig. 1:**
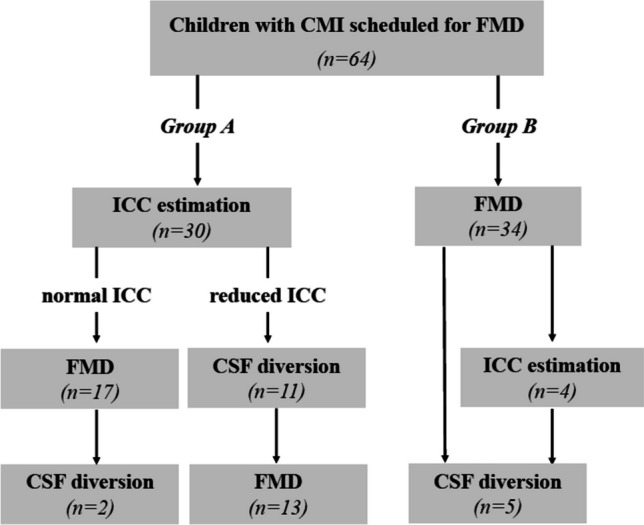
A flowchart retrospectively illustrating the different treatment strategies in the two groups of children with CMI, as analyzed in the present study

In this period, management of pediatric CMI followed two different approaches: (1). Symptomatic pediatric patients with CMI referred to surgery, in whom clinical and/or radiological signs suggested reduced ICC, underwent preoperative overnight ICP measurement with the estimation of ICC from the MWA scores (Group A). If the ICC was abnormally reduced (i.e., MWA abnormally elevated), we first performed cerebrospinal fluid (CSF) diversion surgery (i.e., the ventriculoperitoneal shunt) before FMD, while FMD was the primary treatment if the ICC was normal; (2). Symptomatic children with CMI and more typical clinical presentation without clinical and/or radiological suspicion of reduced ICC underwent FMD as a primary treatment, without any preoperative estimation of ICC (Group B) (Fig. 1).

### Estimation of reduced versus normal ICC

The method of estimating ICC utilized in the present study has been described in detail previously [15], and is therefore only shortly commented here: ICC was estimated from the continuous overnight MWA measurements. For this, an ICP sensor (Codman MicroSensor ICP Transducer; Integra LifeSciences, Princeton, NJ, USA) was placed about 1 cm in the brain parenchyma through a burr hole placed in front of the right coronal suture. The ICP waveform was continuously sampled at 100–200 Hz; the ICP waves were identified, and the pulsatile ICP was expressed as the mean ICP wave amplitude (MWA), utilizing dedicated software (Sensometrics Software; dPCom, Oslo, Norway). From our experience and given the previously shown association between MWA and ICC [16, 18], we considered MWA > 5 mmHg as a surrogate threshold of reduced ICC, and MWA < 5 mmHg of normal ICC [16].

### Surgical treatment

The FMD was always conducted including duraplasty in the following fashion: in a park-bench position with the right side up and the head fixated in a Mayfield clamp in slight flexion, the foramen magnum was exposed by midline dissection, and opened by a C1 laminectomy and a suboccipital craniectomy extending 1.5 cm above the level of the opisthion. Following the resection of the posterior atlantooccipital membrane and opening of the dura with a Y-shaped incision, the microsurgical subarachnoid dissection of the cerebellar tonsils was performed. The caudal part of the fourth ventricle was inspected to see that the foramen of Magendie and the central canal of the medulla were not blocked by arachnoid adhesions. Coagulation or subpial resection of cerebellar tonsils was conducted only optionally, if the tonsils were found to be too voluminous to achieve sufficient decompression even with duraplasty. The dura mater was closed with a watertight 5/0 closure with duraplasty using a triangle-shaped artificial dura graft (Neuro-Patch®; Aesculap, B.Braun, Melsungen, Germany, or Durepair™ Regeneration Matrix, Medtronic, USA). The dural suture was then secured by fibrin sealant (Tisseel; Baxter Healthcare Corporation, Westlake Village, CA, USA) and TachoSil (Baxter, CA, USA), before closing the wound in anatomical layers.

The children with significantly reduced ICC were primarily treated with surgical diversion of CSF, i.e., the ventriculoperitoneal (VP) shunt, to alleviate the increased pulsatile ICP before FMD. We utilized the Codman Hakim® programmable valve or OSV II valve (both Integra LifeSciences, Princeton, NJ, USA). FMD in all these patients was then performed typically a few months after shunting.

### Follow-up

To assess the clinical outcome, an independent investigator (E.B.) not involved in the treatment interviewed all patients/parents by phone and scored the outcome according to Chicago Chiari Outcome Scale (CCOS). This recently proposed [2] externally validated [4, 54] scoring tool uses four postoperative outcomes categories: (a) pain symptoms (mechanical headache, neck/shoulder pain, dysesthesia in the extremities), (b) non-pain symptoms (dysphagia, ataxia, vertigo, muscle weakness, sensory loss, tinnitus, paresthesias, drop attacks), (c) functionality (ability to attend work/school or usual daily responsibilities), and (d) complications to surgery (wound infection, chemical meningitis, CSF leak), graded from 4 (complete resolution) to 3 (persistent but improved), 2 (unchanged) to 1 (worsened), for a total best possible score of 16. CCOS was designed for retrospective chart review and validated for children. It may also be used in a modified version excluding functionality (mCCOS), which not always is exclusively related to CMI, giving the best possible score of 12 [1].

The radiological findings before and after the surgical treatment were retrospectively reviewed by a board-certified neuroradiologist (B.W.). The following aspects were recorded from available MRI sequences obtained in closest proximity to surgery and clinical follow-up, respectively: the extent of tonsillar ectopy below the McRae’s (basion-opisthion) line, presence of syringomyelia and ventriculomegaly (Evans Index > 0.3), macrocephaly, evidence of crowding and medullary compression in the foramen magnum, craniocervical junction (CCJ) anomalies, and occult spinal cord tethering (low lying medullary conus and/or “fatty” filum terminale). Postoperatively, enlargement of the space in the foramen magnum and resolution of the syrinx were noted.

### Statistical analysis

Statistical analyses were performed with SPSS software (version 28.0 for Windows; IBM Corp.). Differences between groups were determined by an independent-sample *t* test for continuous data and a Pearson’s chi-squared test for categorical data. Statistical significance was accepted at the 0.05 level (two-tailed).

## Results

### Patient material

The patient cohort consisted of 64 children (28 boys and 36 girls; mean age 11.1 ± 4.3 years) with CMI treated with FMD during the study period. ICC was preoperatively estimated in 30 (Group A) and not defined in the remaining 34 children (Group B).

### Clinical and radiological differences between the groups

Compared between the groups, we found a significantly higher age (*p* = 0.008) as well as higher frequency of headache and dizziness (both *p* = 0.007), fatigue (*p* = 0.035), and nausea (*p* = 0.047) among patients in Group A. The frequency of other symptoms and radiological findings (in descending order: tonsillar ectopy, crowding in the foramen magnum, syringomyelia, medullary compression, CCJ anomalies, macrocephaly, ventriculomegaly, and spinal cord tethering) was not significantly different between the groups (Table 1).

**Table 1:** Clinical and radiological differences between the groups of children with and without preoperatively estimated ICC, respectively

Parameter		All patients (*n* = 64)	Group A (*n* = 30)	Group B (*n* = 34)	*p*
*Gender (M/F)*		28/36	13/17	15/19	ns
*Age (years)*		11.1 ± 4.3	12.6 ± 3.6	9.8 ± 4.5	0.008
*Symptoms*	*Headache* (%)	45 (70)	26 (87)	19 (56)	0.007
	*Nausea* (%)	18 (28)	12 (40)	6 (18)	0.047
	*Fatigue* (%)	16 (25)	11 (37)	5 (15)	0.035
	*Scoliosis (%)*	16 (25)	6 (20)	10 (29)	ns
	*Dizziness* (%)	14 (22)	11 (37)	3 (9)	0.007
	*Cervical pain* (%)	12 (19)	8 (27)	4 (12)	ns
	*Motor* (%)	10 (16)	3 (10)	7 (21)	ns
	*Sensory* (%)	10 (16)	3 (10)	7 (21)	ns
	*Unsteadiness* (%)	8 (13)	4 (13)	4 (12)	ns
	*Dysphagia* (%)	8 (13)	2 (7)	6 (18)	ns
	*Cognitive* (%)	8 (13)	5 (17)	3 (9)	ns
	*Pain in extremities* (%)	6 (9)	4 (13)	2 (6)	ns
	*Sleep apnea* (%)	6 (9)	3 (10)	3 (9)	ns
	*Dysesthesia* (%)	3 (5)	-	3 (9)	ns
	*Dysphonia* (%)	3 (5)	1 (3)	2 (6)	ns
	*Diplopia* (%)	3 (5)	3 (10)	-	ns
	*Dysarthria*	3(5)	2 (7)	1 (3)	ns
	*Dyspnea* (%)	1 (2)	1 (3)	-	ns
	*Vision* (%)	1 (2)	-	1 (3)	ns
	*Syncope* (%)	1 (2)	-	1 (3)	ns
*Radiology*	*Tonsillar ectopy (mm)*	14.6 ± 4.3	14.3 ± 4.4	14.7 ± 4.3	ns
	*FM crowding (%)*	45/64 (70)	22/30 (73)	23/34 (68)	ns
	*Syringomyelia (%)*	34/62 (55)^1^	15/29 (52)^1^	20/33 (61)^1^	ns
	*Medullary compression (%)*	28/64 (44)	15/30 (50)	13/34 (38)	ns
	*CCJ anomalies (%)*	25/64 (39)^2^	11/30 (37)	14/34 (42)	ns
	*Macrocephaly*	14/64 (22)	4/30 (13)	10/34 (29)	ns
	*Ventriculomegaly (%)*	7/64 (11)	3/30 (10)	4/34 (12)	ns
	*Spinal cord tethering (%)*	2/62 (3)^1^	-	2/33 (6)^1^	ns

*CCJ*, craniocervical junction; *CCOS*, Chicago Chiari Outcome Scale; *FMD*, foramen magnum decompression; *ICC*, intracranial compliance; *ns*, non-significant

Quantitative data in mean ± standard deviation, comparison between subgroups with independent *t* sample test; categorical data in absolute numbers and percent, comparison between subgroups with Pearson’s chi-squared test

^1^data missing in two patients (preoperative spinal MR not available)

^2^dorsal dens angulation (< 80°) in *n* = 22, basilar invagination in *n* = 5, C0/C1 fusion in *n* = 4, platybasia in *n* = 3, condylar/atlas hypoplasia in *n* = 1, C1 lamina aplasia in *n* = 1, failure of posterior midline fusion of C1 lamina 1 *n* = 1 and C1/C2 subluxation in *n* = 1

A preoperative ophthalmological investigation with fundoscopy was available in only 17/30 patients in Group A and 4/34 patients in Group B; without finding papilledema in any of the patients.

### Surgical treatment

In Group A, the mean ICP was 9.3 mmHg ± 3.6, and the mean wave amplitude MWA was 5.3 mmHg ± 1.1. The finding of significantly reduced ICC resulted in an indication for CSF diversion (ventriculoperitoneal shunt) before FMD in 11 children (including eight of them without a finding of papilledema on fundoscopy). Two other patients in this group received shunt after FMD due to CSF-related complications (see below), despite the fact that they had normal ICC before FMD. In total, 13 patients (43%) received VP shunt in this group.

In Group B, all children underwent FMD as a first-line treatment, but five of them (15%) required shunt after FMD due to postoperative hydrocephalus or CSF leakage.

The total number of all surgical procedures related to the treatment of CMI and complications (ICP measurement, FMD, shunt, re-do FMD, shunt revisions) was 2.6 ± 0.9 per patient in Group A, and 1.5 ± 1.1 in Group B (*p* < 0.001; Table 2).

**Table 2:** Details on treatment and outcome data between the groups of children with and without preoperatively estimated ICC, respectively

Treatment	All patients (*n* = 64)	Group A (*n* = 30)	Group B (*n* = 34)	Significance
*ICP implantations*	34	30	4	-
*FMD*	64	30	34	-
*Re-do FMD*	3	1	2^1^	ns
*VP shunt*	18 (28)	13 (43)^2^	5 (15)	0.011
*Filum terminale clipping*	2 (3)	-	2 (6)	**-**
*C1/C2 fixation*	1 (2)	-	1 (3)	**-**
*Fenestration of syrinx*	1	-	1	**-**
*Shunt-related revision*	4	4	-	**-**
*Wound resuture due to CSF leak*	1	-	1	**-**
*Total number of procedures*	131	80	52	
*per patient*	2.1 ± 1.2	2.6 ± 0.9	1.5 ± 1.1	< 0.001
**Outcome**				
*Follow-up length (months)*	83 ± 57	74 ± 45	90 ± 66	ns
Chicago Chiari Outcome Scale (CCOS)				
*Pain symptoms*	3.4 ± 0.7	3.3 ± 0.7	3.4 ± 0.6	ns
*Non-pain symptoms*	3.3 ± 0.8	3.3 ± 0.8	3.3 ± 0.8	ns
*Functionality*	3.4 ± 1.1	3.5 ± 1.0	3.4 ± 1.1	ns
*Complications*	3.6 ± 0.7	3.7 ± 0.6	3.5 ± 0.8	ns
Total CCOS	13.7 ± 1.9	13.8 ± 1.9	13.6 ± 2.0	ns
Total mCCOS	10.3 ± 1.3	10.4 ± 1.5	10.2 ± 1.1	ns
**Radiology**				
*FM space enlargement (%)*	59/64 (92)	28/30 (93)	31/34 (91)	ns
*Syrinx resolution (%)*	33/35 (94)	14/15 (93)	19/20 (95)	ns
**Complications** ***to FMD*** *(%)*	13/64 (20)^3^	2/30 (7)	11/34 (32)	0.011

*CCOS*, Chicago Chiari Outcome Scale; *FMD*, foramen magnum decompression; *ICC*, intracranial compliance; *ICP*, intracranial pressure; *ns*, non-significant

^1^In one of these patients, two re-do FMDs were performed

^2^Before FMD (based on reduced ICC) in* n* = 11, and after FMD in two other patients who had normal ICC before FMD

^3^Fourteen events in 13 patients: need for VP shunt after FMD in *n* = 7 (out of which five in group B); wound infection in *n* = 2, wound leakage/pseudomeningocele in *n* = 3, transient hydrocephalus and C1/C2 subluxation in one patient each, respectively

### Complications

The occurrence of complications related to CSF (including subcutaneous/submuscular CSF accumulation, CSF leakage from the wound, and postoperative hydrocephalus) was significantly lower in Group A (7%) compared to Group B (27%; *p* = 0.036). Along with other complications of FMD seen in Group B, a total of 32% of patients in this group experienced complications after FMD (Table 2).

In Group A, additional complications related to shunting occurred in 4/13 patients (31%): shunt revisions in two patients, leading to shunt explantation in one of them; abdominal hernia at the shunt insertion site in one patient, and overdrainage requiring implantation of the antisiphon device in one patient. There were no complications related to invasive ICP measurements.

### Clinical and radiological follow-up

After a mean follow-up of 83 ± 57 months, CCOS for the whole series was 13.7 ± 1.9 and mCCOS 10.3 ± 1.3. There was no significant difference in CCOS or mCCOS scores between the groups, nor in any of the subcategories (Table 2). We were not able to reach seven patients for a phone interview in January/February 2023. For these patients, CCOS was scored according to information from the latest contact at the outpatient clinic, as noted in their medical charts.

Radiologically, enlargement of CSF space in the foramen magnum after FMD was achieved in 92% and resolutions of syringomyelia in 94% of children, without significant difference between the groups.

## Discussion

**Fig. 2:**
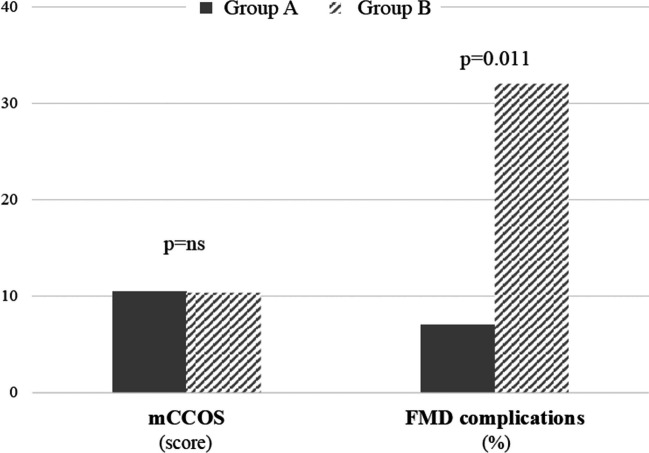
A comparison of outcome scores from modified Chicago Chiari Outcome Scale (mCCOS) and frequency of complications after FMD between cohorts of children in whom intracranial compliance (ICC) was (Group A) and was not (Group B) estimated before FMD (significance levels determined by Pearson’s chi-squared test)

The present observations show that the CSF diversion was required in 43% of symptomatic children with CMI presenting with clinical and/or radiological signs indicative of reduced ICC, in whom ICC was objectively estimated from overnight ICP measurement. In contrast, 15% of children without these signs and no estimation of ICC before FMD still required shunt due to CSF-related complications after FMD. Moreover, the complication rate after FMD was significantly lower in the group of children with a preoperative estimation of ICC, while clinical outcomes were comparable (Fig. 2).

### Intracranial compliance and its significance in CMI

The role of ICC in the pathophysiology of CMI has been previously scarcely explored [13, 20, 22], not least because it is difficult to assess ICC in a clinically feasible and useful fashion. However, reduced ICC may be a clinically relevant pathophysiological mechanism in patients with CMI: The ectopy of cerebellar tonsils and resulting obstruction in the foramen magnum leads to decoupling of intracranial from intraspinal space [20, 30], which appears critical given the fact that intraspinal compartment accounts for two-thirds of the compliance provided by the whole CSF space [47].

Intracranial compliance (ICC) refers to the intracranial pressure–volume reserve capacity [19]. Direct estimation of ICC has received limited attention among neurosurgeons since it requires ICP measurements during the artificial addition or subtraction of an intracranial volume, which is too invasive to be used clinically [19, 26]. As an alternative, we have used ordinary ICP monitoring with an algorithm for the identification of the cardiac-induced single pressure waves to provide an estimate or surrogate marker of ICC. The metric MWA, in contrast to the traditional mean ICP, was previously found to be associated with the direct measurement of ICC; an average of MWA > 4–5 mmHg during the overnight monitoring was accompanied by direct measures of reduced ICC [16, 18]. We have therefore used the MWA score as a surrogate marker of ICC, with the advantage that MWA is measured continually together with mean ICP and thereby provides an online estimation of ICC. For the overall assessment of the individual patient, we defined a threshold for average scores of MWA during overnight monitoring to assess the presence of normal or reduced ICC [24].

### Clinical and radiological indices of reduced ICC

As of today, there are no reliable clinical or radiological parameters that may non-invasively verify or rule out reduced ICC. Among children referred to the preoperative estimation of ICC in the present study (Group A), pressure headache, nausea, fatigue, and dizziness were the dominating symptoms raising the clinical suspicion of abnormal ICC. The symptom burden was therefore—as expected—higher in Group A compared to Group B (Table 1). However, still only 43% of patients in Group A required CSF diversion. On the other hand, 15% of the patients in Group B experienced postoperative complications requiring shunt, despite no initial suspicion of reduced ICC. These observations suggest that it is difficult to predict ICC from clinical and radiological measures alone, and support our practice of estimation of ICC from MWA measurements in symptomatic children with CMI and suspected reduced ICC.

As to radiology, there is no association between reduced ICC and the size of cerebral ventricles [14, 44], particularly in conditions like CMI and idiopathic intracranial hypertension (IIH) [22]. The previously reported frequency of preoperative or postoperative hydrocephalus (in the meaning of radiologically overt ventriculomegaly) in CMI of only 7–10% [37, 49] and 5.1% [6], respectively, is therefore probably an underestimation of the real proportion of patients with reduced ICC [20, 22]. Also in our study, we could not find any relevant radiological parameters that would help identify those with reduced ICC.

**Fig. 3:**
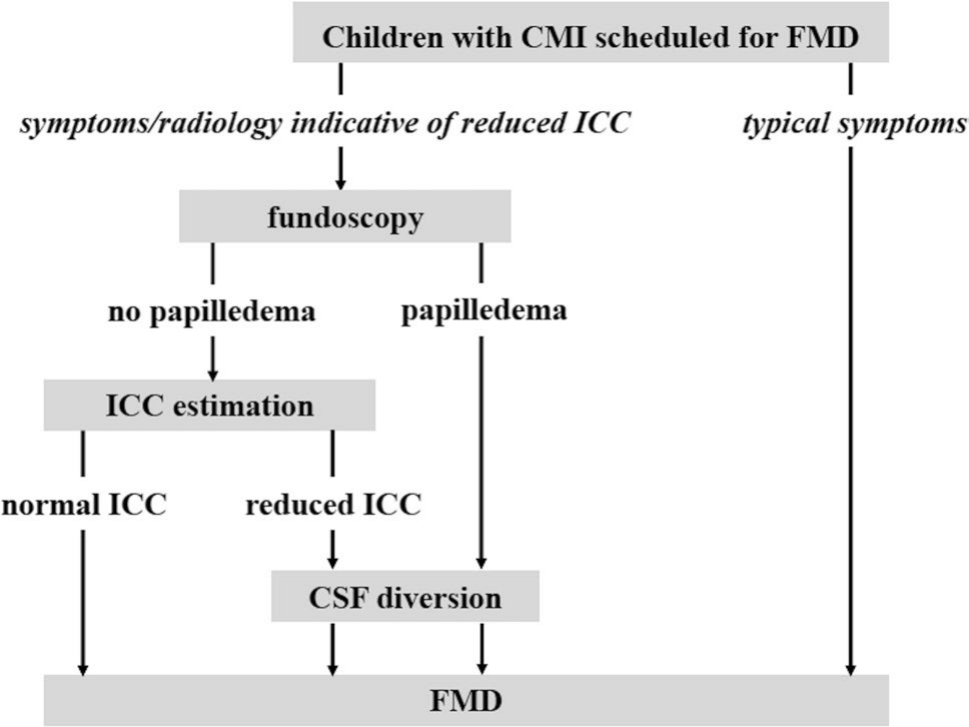
A suggested protocol for preoperative workup of children with CMI referred to FMD, based on the presence of clinical and radiological signs indicative of reduced ICC, as opposed to those presenting with more typical symptoms of CMI

It is known that the finding of papilledema on fundoscopy correlates well with reduced ICC (i.e., abnormally elevated MWA) [31], suggesting that such finding alone can give an indication for CSF diversion without the need for ICP measurement. However, while ICC usually is reduced when papilledema is present [17], the absence of papilledema per se does not exclude reduced ICC. In the present series of patients, the ophthalmologic investigation was available in only approximately one-third of the patients (21 out of 64), but did not show papilledema in any of them, including those (*n* = 8) in whom ICC was found to be significantly reduced and who required shunting. In addition, the incidence of true papilledema among children referred to ophthalmological investigation with suspected papilledema based on fundus examination generally is very low [32]. Thus, the only practical benefit of fundoscopy would be to identify the children with true papilledema, in whom further invasive estimation of ICC is not necessary [38] before indication for CSF diversion (Fig. 3). Finally, as none of the children in our study requiring shunt had papilledema, it is also dubious whether these could be described as suffering from idiopathic intracranial hypertension (IIH), where papilledema is present in majority of patients and where significant tonsillar ectopy can also be found as a secondary phenomenon [7, 22].

There are several different pathophysiological mechanisms possibly associated with CMI: anomalies of craniocervical junction such as cranial settling, tethering of the spinal cord, intraspinal hypotension, cranial constriction, and intracranial hypertension with or without hydrocephalus [23, 37]. In principle and also in our experience, the latter two mechanisms are most frequently associated with primarily reduced ICC, leading to the development of tonsillar ectopy as a secondary event. In the former three mechanisms, ICC may also be found to be reduced, but then most probably secondary to obstruction of CSF flow through the foramen magnum, i.e., where CMI is a primary pathological finding.

### Complications of FMD

According to the existing literature, the most common complications of FMD with duraplasty are those related to CSF circulation: subcutaneous/submuscular CSF accumulation (“pseudomeningocele”), CSF leakage from the wound, or postoperative hydrocephalus. In the pediatric population with CMI, these events—though not always recognized as “complications”—are reported to occur in 8.6 to 19.4% of cases [5, 8, 11, 25, 33]. In our study, these complications occurred in 15.6%, but in only 7% of the patients in Group A compared to 27% in Group B (*p* = 0.036). Our interpretation is that the preoperative estimation of ICC was instrumental in selecting the children who could benefit from CSF diversion surgery before FMD, thus preventing complications to FMD originating from untreated intracranial hypertension and/or reduced ICC.

On the other hand, due to the high percentage of shunts in Group A, the rate of shunt-related complications was naturally also higher in this group, as was the total number of interventions per patient. An important question is whether a higher number of interventions could be justified by reducing the rate of complications after FMD: In our view, it is acceptable, as complications to FMD also represent a significant risk to patients together with potentially suboptimal outcomes after FMD. Moreover, the total number of procedures did not seem to affect the CCOS scores.

The percentage of patients requiring CSF diversion (28%) in treating CMI is quite high in the present series, raising the question of the potential lifelong consequences and psychosocial burden of shunting in these children. However, as the patients in our practice always underwent FMD after initial shunting, providing a potential for gradual normalization of CSF flow and the ICC, they do not tend to present with clinically significant shunt dysfunction. On the other hand, in children treated with a shunt before FMD and later presenting with recurrent tonsillar ectopy and/or syringomyelia, the shunt should always be revised first.

The favorable effect of the endoscopic third ventriculostomy (ETV) as an alternative to VP shunt in patients with CMI and ventriculomegaly has been reported in a few studies, each from a relatively modest number of patients [10, 29, 35, 45, 53]. However, as only three out of 18 patients (17%) who required shunt in our series had radiologically detectable ventriculomegaly (i.e., Evans ratio > 0.3), ETV could possibly be considered in only those few patients, with the same burden regarding the total number of procedures.

### Clinical outcome

It is challenging to objectively assess clinical outcomes after treatment of CMI, particularly in children, as the most common symptoms in CMI are not exclusively specific to this diagnosis, and the clinical presentation often is complex. The methods used in the literature have been very different, making it difficult to compare results across studies [28]. The Chicago Chiari Outcome Scale (CCOS) has since its first description [2] been used in an increasing number of studies [8, 9, 24, 25, 33, 34, 36, 41, 43, 52, 55] and has been externally validated [4, 54]. We found it easy and reliable to use, though with some limitations:

First, it may be challenging to distinguish between symptoms (both “pain” and “non-pain”) specifically related to CMI in cases with more complex clinical presentation.

Second, in the “functionality” part of this scoring tool, particularly when scored after a long follow-up as in our study, the outcome may be significantly influenced by more complex health and social issues, not even related to CMI. For that reason, we chose to score the outcome in our patients according to modified CCOS (mCCOS), in which this part is omitted [1]. Thus, we avoided the situations where some patients with full functionality (i.e., school or work attendance) despite persisting clinical symptoms scored higher than others who improved from their CMI symptoms but did not reach full functionality for unrelated reasons.

Third, in the original description of CCOS [2], it is stated that patients who had persistent pseudotumor cerebri that required medical and/or surgical intervention to improve, such as repeated lumbar punctures or shunt placement, should receive a score of 2. Thus, we gave this score to those patients in both groups who received VP shunt after FMD. However, it appears that the authors of the original CCOS considered shunting to be a “complication” regardless of its reason and timing, which in our opinion not always would be correct: in fact, by preoperative ICC estimation and CSF diversion before FMD, we prevented these complications, and such patients should therefore not have lower complication score in CCOS.

In our series, we achieved very good overall CCOS scores within the range referred to in similar studies from the pediatric population (13.5–15.1) [8, 25, 27, 36, 50, 51, 55]. Also the mCCOS scores were similar to those reported previously [1]. The higher percentage of patients in Group A who received VP shunt had therefore no negative impact on the clinical outcome.

Taken together, we consider it a significant finding that the occurrence of CSF-related complications after FMD may be prevented, provided that children with symptoms indicative of abnormally reduced ICC are recognized during the preoperative workup, and their ICC objectively estimated from ICP measurement (Fig. 3). In particular, we believe that these patients should be considered for CSF diversion (VP shunt) before FMD. This applies particularly to the subgroup of children with CMI presenting with more diffuse complaints such as pressure headache, dizziness, fatigue, and nausea, as opposed to children with more typical symptoms of CMI, where routine preoperative estimation of ICC using the metric MWA appears unnecessary.

### Limitations

The main limitations of the study are its retrospective fashion as well as an obvious selection bias as to which children were referred to the estimation of ICC before FMD and which were not, according to the subjective and investigator-dependent clinical and/radiological indicators of reduced ICC.

Moreover, since not all children in this series routinely underwent preoperative estimation of ICC, it is impossible to know how the ICP scores would compare between the groups.

## Conclusion

In symptomatic children with CMI, we found the preoperative estimation of ICC from the overnight measurement of MWA more reliable for identifying those with reduced ICC than clinical and radiological assessment alone. When children with abnormally reduced ICC were identified and treated with CSF diversion before FMD, the complication rate after FMD was significantly reduced.

## Data Availability

Data or information needed to reproduce the findings presented are available from the corresponding author upon reasonable request.
